# Effect of interleukin-1β on spinal cord nociceptive transmission of normal and monoarthritic rats after disruption of glial function

**DOI:** 10.1186/ar2756

**Published:** 2009-07-08

**Authors:** Luis Constandil, Alejandro Hernández, Teresa Pelissier, Osvaldo Arriagada, Karla Espinoza, Hector Burgos, Claudio Laurido

**Affiliations:** 1 Laboratory of Neurobiology, Department of Biology, Faculty of Chemistry and Biology, University of Santiago of Chile, Ave. Libertador B. O'Higgins 3363, Casilla 40 Correo 33, Santiago, Chile; 2 Program of Molecular and Clinical Pharmacology, Institute of Biomedical Sciences (ICBM), Faculty of Medicine, University of Chile, Independencia 1027, P.O. Box 70000 Santiago 7, Santiago, Chile; 3 School of Psychology, Las Americas University, Ave. Libertad, 1348, Viña del Mar, Valparaiso, Chile

## Abstract

**Introduction:**

Cytokines produced by spinal cord glia after peripheral injuries have a relevant role in the maintenance of pain states. Thus, while IL-1β is overexpressed in the spinal cords of animals submitted to experimental arthritis and other chronic pain models, intrathecal administration of IL-1β to healthy animals induces hyperalgesia and allodynia and enhances wind-up activity in dorsal horn neurons.

**Methods:**

To investigate the functional contribution of glial cells in the spinal cord nociceptive transmission, the effect of intrathecally administered IL-1β was studied in both normal and adjuvant-induced arthritic rats with or without glial inhibition. Four weeks after induction of monoarthritis, rats were treated with the glial cell inhibitor propentofylline (10 μg i.t. daily during 10 days) and submitted to a C-fiber-mediated reflex paradigm evoked by single and repetitive (wind-up) electric stimulation.

**Results:**

Both the propentofylline treatment and the monoarthritic condition modified the stimulating current required for threshold activation of C reflex responses. Intrathecal IL-1β increased spinal cord wind-up activity in normal and monoarthritic rats without propentofylline pre-treatment, but resulted in decreased wind-up activity in normal and monoarthritic propentofylline-treated animals. Intrathecal saline did not produce any effect. Thus, glial inactivation reverted into inhibition the excitatory effect of IL-1β on spinal cord wind-up, irrespective of the normal or monoarthritic condition of rats.

**Conclusions:**

The results suggest that the excitatory effect of nanomolar doses of IL-1β on spinal wind-up in healthy rats is produced by an unidentified glial mediator, while the inhibitory effects of IL-1β on wind-up activity in animals with inactivated glia resulted from a direct effect of the cytokine on dorsal horn neurons. The present study failed to demonstrate a differential sensitivity of normal and monoarthritic rats to IL-1β administration into the spinal cord and to disruption of β glial function, as both normal and monoarthritic animals changes wind-up activity in the same direction after propentofylline treatment, suggesting that after glial inhibition normal and monoarthritic animals behave similarly relative to the capability of dorsal horn neurons to generate wind-up activity when repeatedly stimulated by C-fibers.

## Introduction

Rheumatoid arthritis remains a major health problem worldwide, with a prevalence that may amount to one case per 100 people depending on the geographical area of the world considered [1]. Among other major impairing health problems associated with rheumatoid arthritis, pain emerges as the most commonly reported and prevalent disabilitating condition, but current therapies are still suboptimal. One reason for this, among other factors, may be that current therapies for rheumatoid arthritis do not include glial cells as a target for the origin and/or maintenance of pain. In this regard, preclinical studies have shown that adjuvant-induced arthritic rats, a widely used animal model of human rheumatoid arthritis, exhibited glial activation with increased mRNA and protein expressions of both IL-1 and TNFα in the spinal cord [2]. Interestingly, disruption of glial activation in these animals by intrathecal injection of the glial metabolic inhibitor fluorocitrate, reversibly suppressed thermal hyperalgesia and mechanical allodynia evoked in arthritic rats [3], pointing to a functional role of upregulated glial products in arthritic pain, such as IL-1 and TNFα.

The role of glial cells in the pathogenesis of chronic pain is beginning to be understood. Following inflammation and damage of peripheral tissues, the spinal cord responds with a robust glial reaction characterized by proliferation, hypertrophy, decreased ramification, and upregulated expression of pro-inflammatory cytokines such as IL-1β and TNF-α. This suggests that some spinal cytokines of glial origin are involved in the central mechanisms underlying the maintenance and exaggeration of pain states [4-7]. Further support to this idea is provided by studies showing that intrathecal administration of IL-1 and TNFα in healthy rodents induces hyperalgesia and allodynia [8-13], and enhances both the acute response and the wind-up activity of dorsal horn neurons [14,15].

In order to study the contribution of glial activation and the associated upregulated expression of IL-1β on spinal cord nociceptive transmission in arthritic rats, we used the compound propentofylline (3,7-dihydro-3-methyl-1-(5-oxohexyl)-7-propyl-1*H*-purine-2,6-dione) to disrupt glial activation. This compound is an ethylxanthine derivative previously found to attenuate astrocytic activation in a rodent model of ischemia [16]. Systemic application of propentofylline has been found to revert thermal hyperalgesia [17] and mechanical allodynia induced by peripheral nerve injury [17,18], while intrathecal administration of propentofylline exhibited antiallodynic properties in rat models of neuropathic pain [19] and attenuated vincristine-induced peripheral neuropathy [20]. Thus, in the current study we examined if propentofylline administration to adjuvant-induced arthritic and healthy control rats could alter the spinal cord nociceptive transmission to single and repetitive (wind-up) stimulation, and modify the pronociceptive effect of intrathecal IL-1β on the electrophysiological parameters. This was carried out by comparing in propentofylline- and saline-treated rats, the effect of intrathecally administered IL-1β on single integrated C-reflex and its effect on the potentiation of the responses evoked by repetitive electric stimulation of the sural nerve receptive field (wind-up). As previously reported, wind-up activity in dorsal horn neurons is a C-fiber-mediated synaptic potentiation phenomenon of particular importance for the development and maintenance of chronic pain [21], but the role of glia and cytokines on wind-up activity in arthritic animals has received little attention.

## Materials and methods

### Animals

This investigation was performed following protocols approved by the Animal Care and Use Committee of the University of Santiago in Chile and was also in accordance with the ethical standards for investigations of experimental pain in animals of The Committee for Research on Ethical Issues of the International Association for the Study of Pain [22]. Experiments were performed in 32 normal (N) and 32 monoarthritic (M) Sprague-Dawley rats weighing 280 to 320 g. Monoarthritis was induced by injecting 0.05 ml of complete Freund's adjuvant into the right tibio-tarsal joint under brief halothane anesthesia. Complete Freund's adjuvant was prepared as described by Butler and colleagues [23]. Control rats were given intra-articular injections (right tibio-tarsal joint) of 0.05 ml of the vehicle used to suspend mycobacteria. Animals were housed five per cage under standard laboratory conditions and were given food and water *ad libitum*. With the purpose of knowing the monoarthritic and hyperalgesic condition of the rats, we measured the circumference of the injected tibio-tarsal joint (from 2.75 ± 0.25 cm [mean ± standard error of the mean] to 4.3 ± 0.3 cm after four weeks) as well as the vocalization threshold (225 ± 12.5 g to 172 ± 13 g after four weeks) to graded paw pressure (Ugo Basile analgesiameter, Comerio VA, Italy).

Four weeks after injecting the tibio-tarsal joint, once a stable vocalization threshold value to graded paw pressure was determined, eight monoarthritic and eight normal rats were given once daily intrathecal injections of 10 μg propentofylline (P) in 10 μl saline for 10 days. This 10-day treatment has been shown to produce glial inhibition, as revealed by a decrease in both CR3/CD11b and glial fibrillary acidic protein, which are microglial and astrocytic activation markers, respectively, and to attenuate hyperalgesia induced by nerve transection in rats [19,24]. Eight monoarthritic and eight normal additional rats receiving intrathecal injections of saline (S) for 10 days served as controls. Thus, the four groups of rats were: NP rats which were normal rats receiving intrathecal propentofylline; NS rats which were normal rats receiving intrathecal saline; MP rats which were monoarthritic rats receiving intrathecal propentofylline; and MS rats which were monoarthritic rats receiving intrathecal saline. All intrathecal injections (10 μl volume) were given to unanesthetized rats by means of direct percutaneous injection at the L5 to L6 interspace using a 0.5 inch 26-gauge hypodermic needle connected to a Hamilton syringe [25], and correct subarachnoid positioning of the tip of the needle was verified by the generation of a tail-flick. Afterwards, at day 11, the animals were submitted to the electrophysiological study. All the experiments were performed blind (LC).

### C-fiber evoked nociceptive reflex

The C-reflex, elicited in the right hindlimb of urethane anesthetized rats (1.2 g/kg intraperitoneally), was recorded as described previously [15,26]. Briefly, rectangular electric pulses of supramaximal strength and 2 ms' duration were applied every 10 seconds to the sural nerve receptive field by means of two stainless steel needles inserted into the skin of toes four and five (Grass S11 stimulator equipped with a Grass SIU 5 stimulus isolation unit and a Grass CCU 1A constant current unit, Astro-Med, Inc., West Warwick, RI, USA). The C-fiber-evoked reflex response was recorded from the ipsilateral biceps femoris muscle by utilizing another pair of stainless steel needles. After amplification (Grass P511 preamplifier; Astro-Med, Inc., West Warwick, RI, USA), the electromyographic responses were digitized at 100 KHz and integrated in a time-window from 150 to 450 ms after the stimulus by a Powerlab ML 820 instrument (ADInstruments, Castle Hill, NSW, Australia). Once stable C-reflex responses were obtained, the stimulus strength was lowered and the current required for threshold activation of the C-reflex determined. The values of current in mA (Table 1) obtained in the different groups of animals (NS, NP, MS, and MP groups) were stored to be analyzed later by means of a two-way analysis of variance (ANOVA; Prism 3.0, GraphPad Software Inc., San Diego, CA, USA). Integrated C-reflex responses, evoked by single stimuli with two times the intensity of the threshold stimulating current, were then recorded. Afterwards, trains of 12 stimuli each at 1 Hz were delivered to the toes in order to develop wind-up activity. In the C-reflex paradigm, wind-up consists of a stimulus frequency-dependent remarkable increment of the electromyographic integrated response [11]. All responses were stored on hard disk for later analysis. Least square regression lines were fitted among experimental points showing only incremental trend (prior to wind-up saturation at the sixth or seventh stimulus), discarding the remaining points (Origin 6.0 software, Microcal Software, Inc., Northampton, MA, USA), as described elsewhere [11]. The slopes of the regression lines represent wind-up scores.

**Table 1:** Stimulating current (mA) required for threshold activation of C-fiber evoked reflex responses in normal and monoarthritic rats treated with propentofylline (10 μg/10 μl daily) or saline (10 μl daily) during 10 days

	Saline treated	Propentofylline treated
Normal	6.3 ± 0.4	8.2 ± 0.5*
Monoarthritic	3.7 ± 0.6^#^	7.5 ± 0.7*

Values are means ± standard error the mean of stimulating current required (in mA) in the NS, MS, NP and MP groups. Two-way analysis of variance (ANOVA) identified the propentofylline treatment (*P *ANOVA < 0.0001, *F *= 25.79) and the monoarthritic condition (*P *= 0.0065, *F *= 8.64) as significant factors influencing the stimulating current required for threshold activation of the C-reflex. No propentofylline treatment × monoarthritic condition interaction was observed (*P *ANOVA = 0.1016, F = 2.87). Significant differences (*P *< 0.01) between propentofylline- and saline-treated groups are denoted by asterisks, while significant differences between monoarthritic and normal groups (*P *< 0.01) are indicated by the superscript # (according to the Bonferroni *post hoc *test). n = 8 animals for each group.

NP = normal rats receiving intrathecal propentofylline; NS = normal rats receiving intrathecal saline; MP = monoarthritic rats receiving intrathecal propentofylline; MS = monoarthritic rats receiving intrathecal saline.

### Data analysis

In all animals the experiments began with the measurement of the current required for threshold activation of the C-reflex in each of four groups of animals. Two-way ANOVA followed by the Bonferroni multiple comparisons test were used to identify the drug treatment (propentofylline) and/or the monoarthritis as factors influencing this parameter in normal and monoarthritic rats treated with propentofylline. Afterwards, a basal recording of both integrated C-reflex responses and wind-up activity prior to the intrathecal administration of recombinant IL-1β (2 ng/10 μl, equivalent to 11.4 nM) or saline (10 μl). This intrathecal dose of IL-1β has been shown to increase C-fiber evoked responses and wind-up activity in spinal cords of normal rats [8,9]. The effects of IL-1β or saline on the integrated C-reflex responses and wind-up scores were assessed 10, 20 and 40 minutes post-injection, and the results expressed as time-course of the percent change induced. Statistically significant effects of IL-1β within groups were identified by one-way ANOVA, followed by the Dunnett multiple comparisons test. To appreciate the global effect of IL-1β on the complete period of testing, the area under curves (AUCs) for both the integrated responses and wind-up activity were calculated from time zero to 40 minutes (period of testing) by using the Microcal Origin 6.0 software (Microcal Software, Inc., Northampton, MA, USA) and plotted in terms of percent variation. Two-way ANOVA followed by the Bonferroni multiple comparisons test were used to identify the drug treatment (propentofylline) and/or the pain model (monoarthritis) as factors influencing the effect of IL-1β on the integrated C-reflex responses and wind-up scores. When a *P *value in the ANOVAs was less than 0.05, the Bonferroni *post-hoc *multiple comparisons test was used with a confidence interval of 95% (Prism 3.0, GraphPad Software Inc., San Diego, CA, USA).

## Results

Application of single constant electric pulses to toes, at 0.1 Hz, evoked C-fiber-mediated reflex responses in the ipsilateral biceps femoris muscle in both normal (N) and monoarthritic (M) rats, with chronic propentofylline (P) or saline (S) pretreatment. The stimulating current required for threshold activation of the C-reflex in each of four groups of animals is shown in Table 1. It can be observed that NS rats required a stimulating current of 6.3 ± 0.4 mA for threshold activation of the C-reflex, while a significantly greater stimulating current of 8.2 ± 0.5 mA (*P *< 0.01) was necessary to evoke threshold C-reflexes in normal-propentofylline (NP) animals. In MS rats the stimulating current required for threshold activation of the C reflex was 3.7 ± 0.6 mA (*P *< 0.01 with respect to NS rats), whereas MP animals required 7.5 ± 0.7 mA (*P *< 0.01 with respect to MS animals).

**Figure 1:**
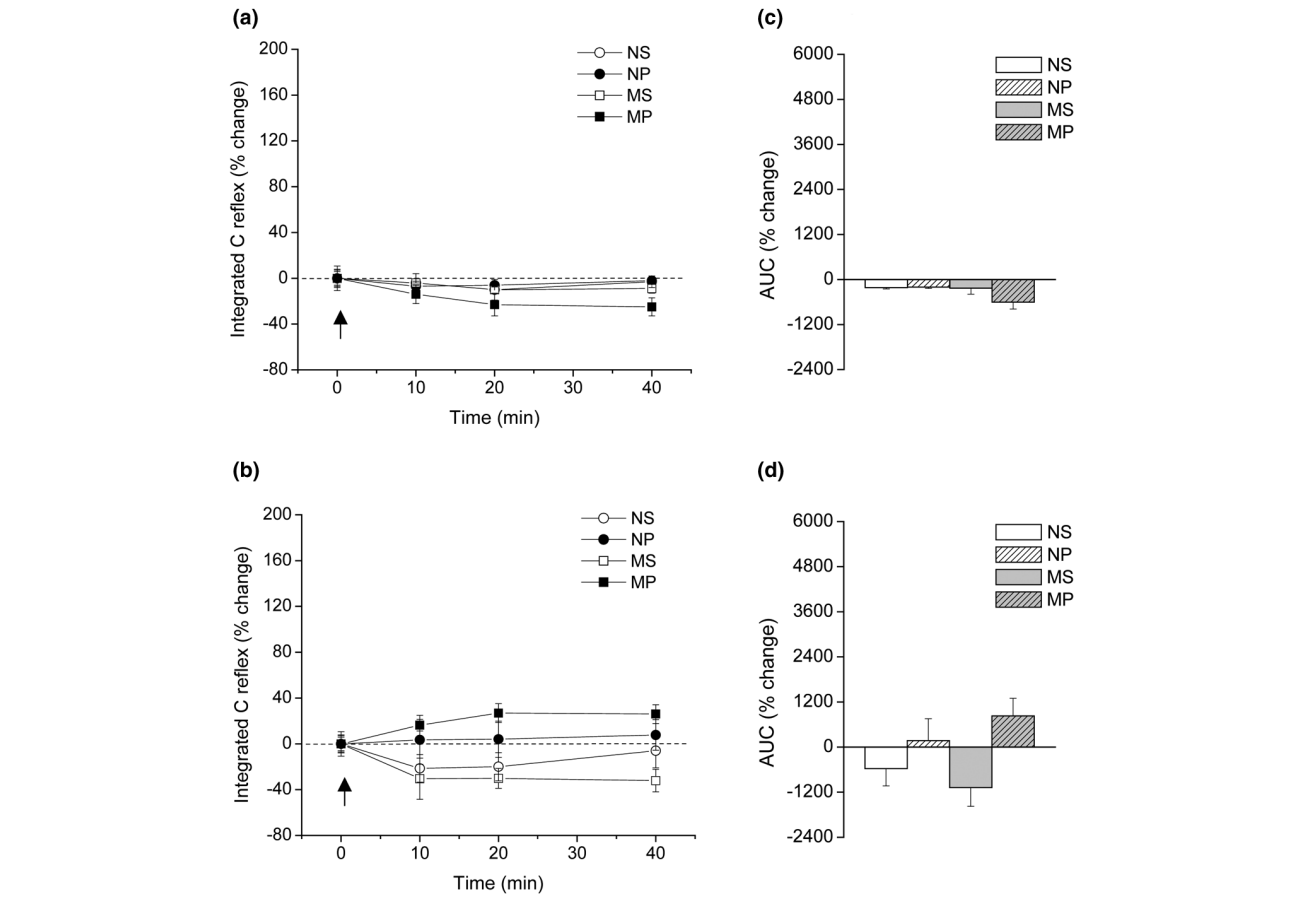
Effect of IL-1β on C-reflex integrated activity in propentofylline-and saline-treated normal and monoarthritic rats (NS, MS, NP, and MP groups). **(a) **Time course of integrated C-reflex responses (% change) 10, 20 and 40 minutes after administration of saline intrathecal. **(b) **Time course of integrated C-reflex responses (% change) 10, 20 and 40 minutes after administration of 2 ng IL-1β intrathecally. The arrow indicates injection of saline or IL-1β at zero time. Values are means ± standard error of the mean (SEM). n = 8 rats in all groups. One-way analysis of variance (ANOVA) did not detect significant intra-group changes in either group after intrathecal saline or after IL-1β. **(c) **Global effect of saline intrathecally and **(d) **2 ng IL-1β intrathecally on integrated C-reflex responses on the 40-minute period of testing, as revealed by percent change of area under the curves (AUCs). Values are means ± SEM. n = 8 rats in all groups. Two-way ANOVA detected that neither the propentofylline-treatment, nor the monoarthritic condition, nor the combination of propentofylline-treatment and monoarthritis affected the AUCs scores significantly or modified the response to saline intrathecally or to IL-1β intrathecally. NP = normal rats receiving intrathecal propentofylline; NS = normal rats receiving intrathecal saline; MP = monoarthritic rats receiving intrathecal propentofylline; MS = monoarthritic rats receiving intrathecal saline.

**Figure 2:**
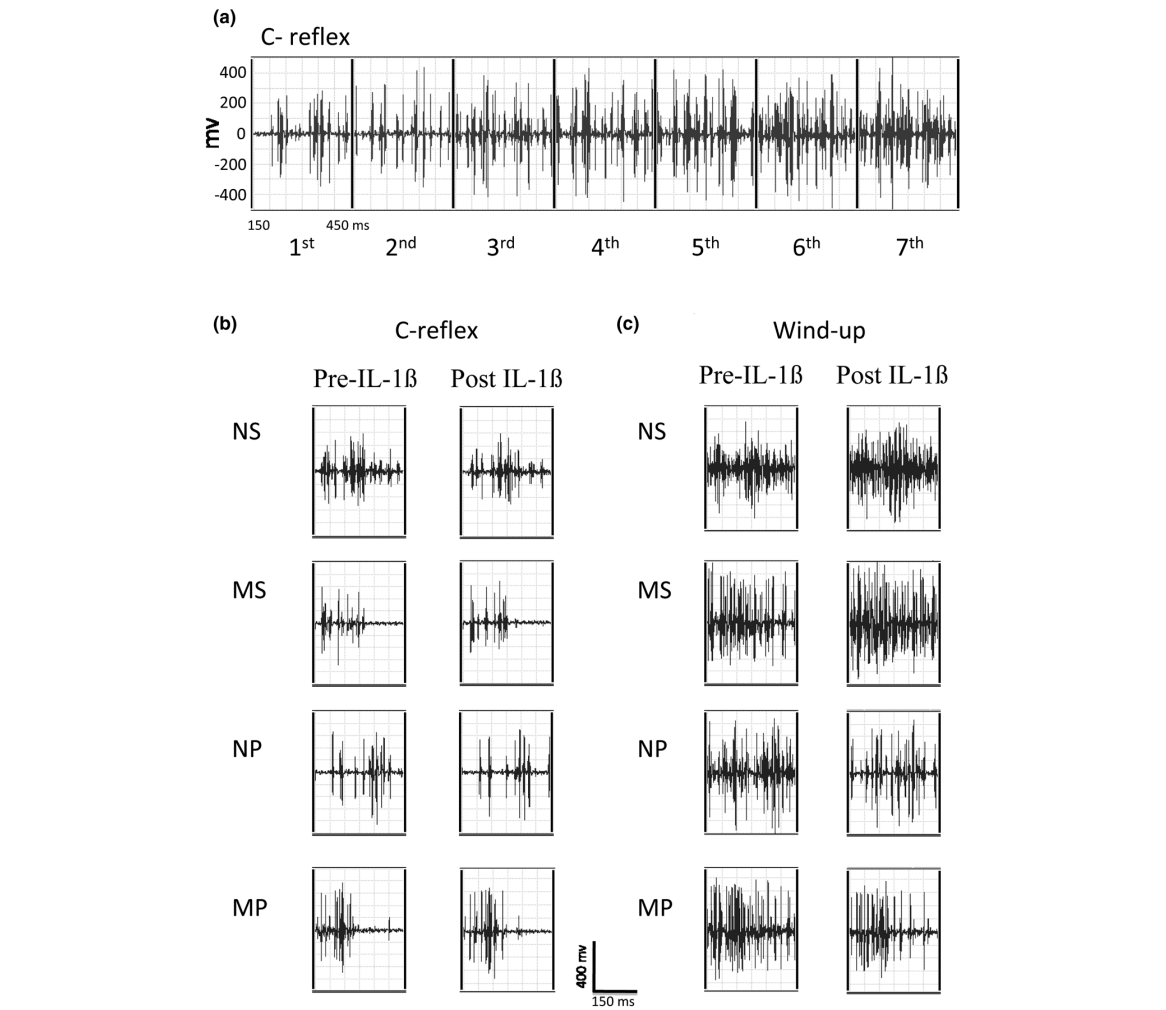
Representative traces showing the effect of a stimulating train and of IL-1β on C-reflex responses. **(a) **Representative traces showing C-reflex potentiation (wind-up) as the stimulating train progresses from the first to the seventh stimulus number. After the seventh stimulus the potentiation reach a plateau and C-reflex response does not grow (not shown). **(b) **Representative traces of C-reflex responses taken from one animal per group (NS, MS, NP, and MP) showing pre-drug traces (left side) and 20 minutes post IL-β traces (right side). **(c) **Representative traces of potentiated C-reflex responses (wind-up) taken from one animal per group (NS, MS, NP and MP): left side = pre-drug traces; right side = 20 minutes post IL-β potentiated traces. Calibration bars are shown at the bottom. NP = normal rats receiving intrathecal propentofylline; NS = normal rats receiving intrathecal saline; MP = monoarthritic rats receiving intrathecal propentofylline; MS = monoarthritic rats receiving intrathecal saline.

Intrathecal administration of a single dose of 2 ng of IL-1β to normal or to monoarthritic rats with or without propentofylline treatment, did not produce significant changes either in the time-course of integrated C-reflex responses (Figure 1b) or in AUCs during the complete 40-minute period of testing (Figure 1d). Intrathecal saline was also ineffective in these respects (Figures 1a and 1c). Representative traces for the effects of IL-1β administration on C-reflex responses are shown in Figure 2b.

**Figure 3:**
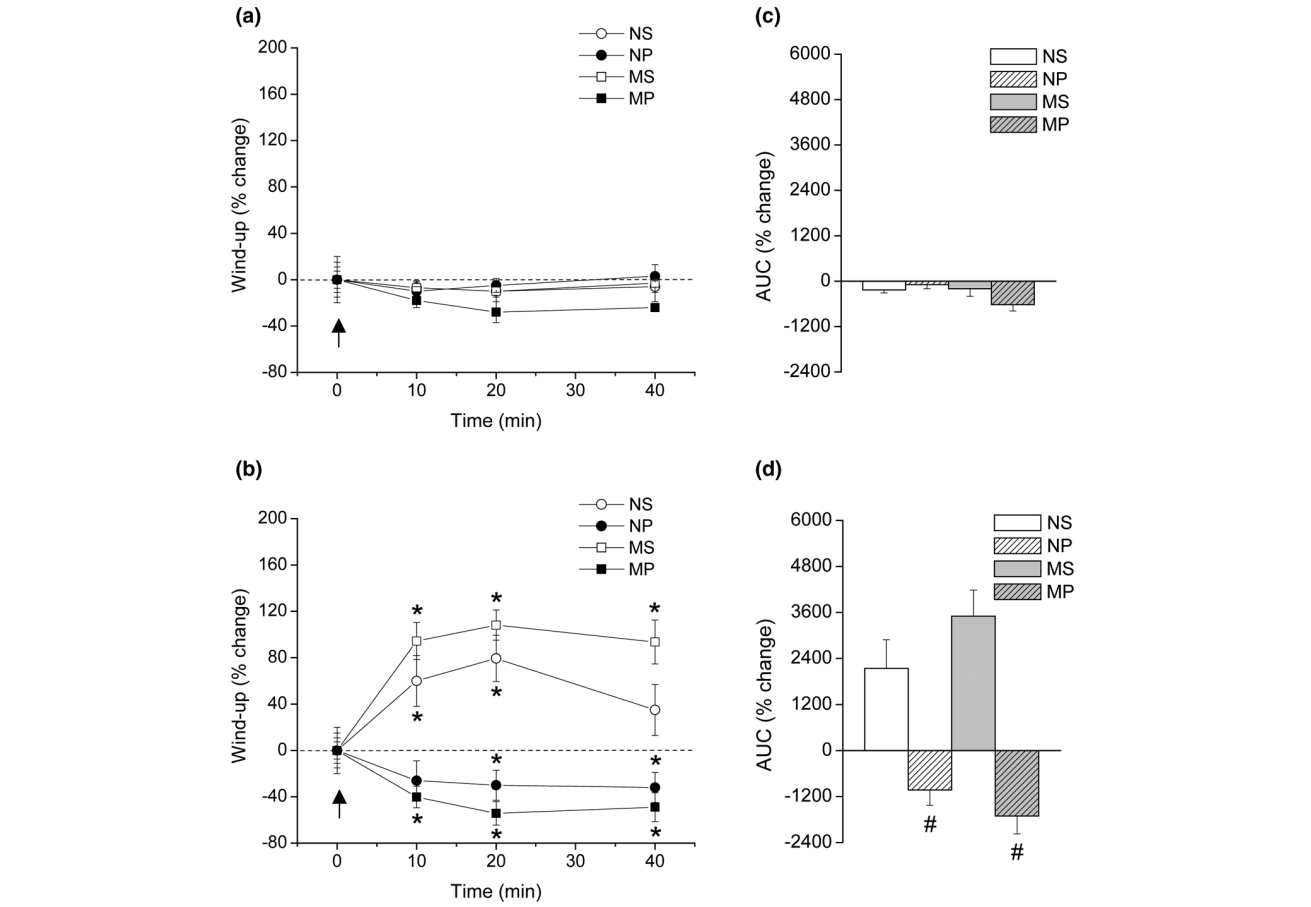
Effect of IL-1β on spinal cord wind-up activity in propentofylline- and saline-treated normal and monoarthritic rats (NS, MS, NP and MP groups). **(a) **Time course of wind-up scores (% change) 10, 20 and 40 minutes after administration of saline intrathecally. One-way analysis of variance (ANOVA) did not detect significant intra-group changes in either group after intrathecal saline. The arrow indicates injection of saline at zero time. Values are means ± standard error of the mean (SEM). n = 8 rats in all groups. **(b) **Time course of wind-up scores (% change) 10, 20 and 40 minutes after administration of 2 ng IL-1β intrathecally. The arrow indicates injection of IL-1β at zero time. Values are means ± SEM. n = 8 rats in all groups. Values are means ± SEM. n = 8 rats in all groups. Intra-group analyzes by one-way ANOVA detected significant wind-up increases in the NS and MS groups after intrathecal IL-1β (NS group: *P *ANOVA = 0.0403, *F *= 3.154; MS group: *P *ANOVA < 0.0004, *F *= 8.363), and significant wind-up decreases in the NP and MP groups after intrathecal IL-1β (NP group: *P *ANOVA = 0.0407, *F *= 3.147; MP group: *P *ANOVA = 0.0135, *F *= 4.253). Significant changes after IL-1β administration are denoted by the asterisk (*P < 0.05, Dunnett *post hoc *test). **(c) **Global effect of saline intrathecally on C-reflex wind-up activity on the 40-minute period of testing, as revealed by percent change of area under the curves (AUCs). Values are means ± SEM. n = 8 rats in all groups. Two-way ANOVA detected that neither the propentofylline-treatment, nor the monoarthritic condition, nor the combination of propentofylline-treatment and monoarthritis affected the AUC scores significantly or modified the response to saline intrathecally. **(d) **Global effect of 2 ng IL-1β intrathecally on C-reflex wind-up activity on the 40-minute period of testing, as revealed by percent change of AUCs. Values are means ± SEM. n = 8 rats in all groups. Two-way ANOVA identified the propentofylline treatment (*P *ANOVA < 0.0001, *F *= 46.91), but not the monoarthritic condition (*P *ANOVA = 0.5799, *F *= 0.31), as a factor influencing the effect of IL-1β on wind-up activity. ^# ^indicates statistically significant difference (*P *< 0.01, Bonferroni *post hoc *test) when comparing propentofylline-treated animals (NP and MP) against the respective saline-treated animals (NS and MS). NP = normal rats receiving intrathecal propentofylline; NS = normal rats receiving intrathecal saline; MP = monoarthritic rats receiving intrathecal propentofylline; MS = monoarthritic rats receiving intrathecal saline.

Application of 12 successive constant electric pulses with two-fold threshold intensity, at 1 Hz, induced spinal wind-up in all groups of rats, as revealed by the gradual but remarkable increase of the integrated C-reflex activity generated by the repetitive stimuli. Figure 2a shows the potentiation of the C-reflex (wind-up) taken from a representative experiment as the stimulating train progresses from the first to the seventh pulse. Intrathecal administration of a single dose of 2 ng of IL-1β to the NS group resulted in about 80% increase of wind-up activity 20 minutes after the injection (Figure 3b, *P *< 0.05). In contrast, 2 ng of IL-1β intrathecally administered to the NP group produced around 30% reduction in wind-up scores 20 to 40 minutes after injection (Figure 3b, *P *< 0.05). Administration of IL-1β intrathecally to monoarthritic rats produced similar effects on wind-up activity to that induced in normal animals (Figure 3b), that is a significant increase (110% increase) of wind-up scores in the MS group but a decrease (55% reduction) of wind-up scores in the MP group 20 and 40 minutes after injection of the cytokine (*P *< 0.05). Intrathecal saline did not produce any significant effect in wind-up of either normal or monoarthritic animals (Figures 3a and 3c). Accordingly, upon analyzing the global effect of IL-1β on wind-up activity during the complete 40-minute period of testing (% change of AUCs), two-way ANOVA identified the propentofylline treatment, but not the monoarthritic condition, as a factor influencing the effect of IL-1β on wind-up activity in both normal and monoarthritic rats (Figure 3d; *P *ANOVA < 0.0001; *P *< 0.01, Bonferroni *post hoc *test). No interaction of the two factors (propentofylline treatment × monoarthritic condition) was detected, meaning that the propentofylline treatment modified in a similar way the wind-up change elicited by IL-1β administration, irrespective the normal or monoarthritic condition of rats. Representative traces for the effects of IL-1β administration on wind-up activity are shown in Figure 2c.

## Discussion

Our results show that in the rat, a 10-day period of treatment with propentofylline intrathecally did not block the ability of dorsal horn neurons to respond to C-fiber nociceptive stimulation and to develop wind-up activity during repetitive C input, but increased the threshold for the triggering of C-fiber-dependent nociceptive reflexes, thus suggesting that glial cells of the spinal cord dorsal horn play some role in pain transmission conveyed by the C-fiber population even in the absence of injury in peripheral sensitive nerves and/or in central spinal cord neurons. On the other hand, adjuvant-induced arthritis decreased the stimulating threshold to evoke C-reflex responses, thus confirming previous observations [11]. Interestingly, intrathecal propentofylline treatment increased the threshold for electrical activation of C-reflexes in monoarthritic rats to values found in normal rats, thus pointing to a role of some spinal glial products in the maintenance of a low excitation threshold for C-reflex activation during arthritis. As it is known that propentofylline affects glial activation and thereby the production of glial proinflammatory cytokines, but it seems propentofylline is unable to produce a direct effect on neurons. The present results also showed that intrathecal administration of IL-1β increased synaptic potentiation to a train of stimuli (wind-up) in the spinal cords of both normal and monoarthritic rats, while not affecting the spinal cord transmission of spinal C-reflex to a single stimulus. This observation suggests that IL-1β of glial origin could play a role in the maintenance of chronic pain by increasing wind-up activity in dorsal horn nociceptive neurons via direct excitation of IL-1 receptors existing in presynaptic afferent terminals and/or second-order neurons [27], or indirectly by acting on glial cells. Interestingly, the present results demonstrated that the intrathecal propentofylline pre-treatment turned the excitatory effect of IL-1β on spinal cord wind-up activity into inhibition, in both normal and monoarthritic rats. This observation suggests the exogenous IL-1β did not act directly on IL-1 receptors of dorsal horn neurons to enhance wind-up activity, but probably on glial IL-1 receptors, thereby inducing the release of a glial mediator responsible for the excitatory effects observed in saline-treated normal and monoarthritic rats. In this respect, there is a variety of potential glial mediators that can fulfill an excitatory role on dorsal horn nociceptive neurons [3]. Firstly, the excitatory amino acid glutamate, which is known to be released from spinal cord glia and play a major role in wind-up elicitation. Second, the ubiquitous molecule nitric oxide, which has been directly implicated in glutamate release from primary nociceptive afferent terminals. Third, other cytokines, such as TNF-α, which have been described as having excitatory activity in dorsal horn cells [12]. Fourth, the glial mediator D-serine, which binds to the glycine site of the NMDA receptor and has been shown to enhance the C-response of dorsal horn neurons [28] and facilitation of the tail-flick reflex [29] in normal rats. All these mediators can potentially be released from glia after glial cell stimulation with IL-1β, provided glial cells are intact.

In contrast, the inhibitory effect of intrathecal IL-1β on wind-up activity in propentofylline-treated rats is probably the result of a direct inhibitory effect of the cytokine on dorsal horn neurons, which would be observed only when glial cells are inhibited by propentofylline. In this regard, inhibitory neuronal effects of IL-1β have been shown in warm-sensitive [30] and glucose-sensitive [31] neurons of the hypothalamus, while both inhibitory and excitatory effects of IL-1β have been observed on neocortical neurons [32]. Rapid (minutes) inhibitory effects of IL-1β on firing rate of hypothalamic neurons have been shown to be dependent on activation of protein kinase Src downstream of the association of the cytosolic adaptor protein MyD88 to the IL-1 receptor [33].

Using patch-clamp techniques it has been demonstrated that at physiologic picomolar concentrations IL-1β exerted excitatory effects on central neurons via activation of a non-selective cationic current, while at pathologic nanomolar levels IL-1β inhibited central neurons by inducing membrane hyperpolarization [34]. Other patch-clamp studies demonstrated that nanomolar concentrations of IL-1β decreased inward calcium depolarizing currents in hippocampal neurons [35] and inward sodium depolarizing currents in retinal ganglion cells [36], which may give a mechanistic support to the inhibitory effect of the intrathecally-administered nanomolar dose of IL-1β on C-reflex wind-up evoked in propentofylline-treated animals. This also may explain the results that show that administration of high intrathecal doses of IL-1β (over 10 ng IL-1 intrathecal) could produce anti-nociception in a rat model of peripheral inflammatory pain [37]. As a whole, the present observations do not support a direct excitatory role for glial IL-1β on the nociceptive processing of spinal cord neurons to repetitive C input but an indirect one via the release of other glial excitatory products (i.e. glutamate), IL-1β being rather involved in the fueling of the glial inflammatory response as part of a glial autocrine loop that may occur during chronic arthritic pain. In these conditions, any direct inhibitory effect of IL-1β on dorsal horn neurons would be exceeded by the excitatory effect of glial excitatory products on neuronal activity, a situation not possible when glia is inhibited by propentofylline.

Finally, the present study failed to demonstrate a differential sensitivity of normal and monoarthritic rats to IL-1β administration into the spinal cord, suggesting that adjuvant-induced arthritis in rat did not result in marked upregulation of glial and/or neuronal IL-1 receptors. However, alternative explanations involving high occupancy of upregulated IL-1 receptors by endogenous IL-1β or by the endogenous IL-1 receptor antagonist which could be highly expressed in monoarthritic rats are also possible. Besides, the present study also failed to demonstrate a differential response of normal and monoarthritic rats after disruption of glial function, at least when the animals were tested to IL-1β challenge, as both normal and monoarthritic animals changes wind-up activity in the same direction after propentofylline treatment. This observation suggests that after glial inhibition, normal and monoarthritic animals behave similarly relative to the capability of dorsal horn neurons to generate wind-up activity when repeatedly stimulated by C-fibers.

## Conclusions

Both the propentofylline treatment and the monoarthritic condition modified the stimulating current required for threshold activation of C-reflex responses. Intrathecal IL-1β increased spinal cord wind-up activity in normal and monoarthritic rats without propentofylline pre-treatment, but resulted in decreased wind-up activity in normal and monoarthritic propentofylline-treated animals. Intrathecal saline did not produce any effect. Thus, glial inactivation reverted to inhibition the excitatory effect of IL-1β on spinal cord wind-up, irrespective of the normal or monoarthritic condition of rats. The results suggest that the excitatory effect of nanomolar doses of IL-1β on spinal wind-up in healthy rats is produced by an unidentified glial mediator, while the inhibitory effects of IL-1β on wind-up activity in animals with inactivated glia might result from a direct effect of the cytokine on dorsal horn neurons. Finally, spinal cord glial inhibition results in decreased potentiation of repetitive nociceptive input, thus suggesting future clinical applications in arthritic pain once glial inhibitors are available for human use.

## Abbreviations

ANOVA: analysis of variance; AUC: area under curve; IL-1β: interleukin-1beta; TNF-α: tumor necrosis factor-alpha.

## Competing interests

The authors declare that they have no competing interests.

## Authors' contributions

LC, OA, and KE performed most of the experiments. TP performed experiments in inducing monoarthritis. LC, AH, TP, HB, and CL conceived the study and participated in the design, in the interpretation of results, and in drafting the manuscript. All authors read and approved the final manuscript.
